# Advances in Materials for Recent Low-Profile Implantable Bioelectronics

**DOI:** 10.3390/ma11040522

**Published:** 2018-03-29

**Authors:** Yanfei Chen, Yun-Soung Kim, Bryan W. Tillman, Woon-Hong Yeo, Youngjae Chun

**Affiliations:** 1 Department of Industrial Engineering, Swanson School of Engineering, University of Pittsburgh, Pittsburgh, PA 15261, USA; yanfeichen@pitt.edu; 2 George W. Woodruff School of Mechanical Engineering, College of Engineering, Georgia Institute of Technology, Atlanta, GA 30332, USA; ysk@me.gatech.edu; 3 Division of Vascular Surgery, University of Pittsburgh Medical Center, Pittsburgh, PA 15260, USA; tillmanbw@upmc.edu; 4 McGowan Institute for Regenerative Medicine, University of Pittsburgh, Pittsburgh, PA 15261, USA; 5 Center for Flexible Electronics, Institute for Electronics and Nanotechnology, Bioengineering Program, Petit Institute for Bioengineering and Biosciences, Neural Engineering Center, Georgia Institute of Technology, Atlanta, GA 30332, USA; 6 Department of Bioengineering, Swanson School of Engineering, University of Pittsburgh, Pittsburgh, PA 15261, USA

**Keywords:** implantable materials, low-profile bioelectronics, micro/nanofabrication, medical devices, biodegradable materials, miniaturization

## Abstract

The rapid development of micro/nanofabrication technologies to engineer a variety of materials has enabled new types of bioelectronics for health monitoring and disease diagnostics. In this review, we summarize widely used electronic materials in recent low-profile implantable systems, including traditional metals and semiconductors, soft polymers, biodegradable metals, and organic materials. Silicon-based compounds have represented the traditional materials in medical devices, due to the fully established fabrication processes. Examples include miniaturized sensors for monitoring intraocular pressure and blood pressure, which are designed in an ultra-thin diaphragm to react with the applied pressure. These sensors are integrated into rigid circuits and multiple modules; this brings challenges regarding the fundamental material’s property mismatch with the targeted human tissues, which are intrinsically soft. Therefore, many polymeric materials have been investigated for hybrid integration with well-characterized functional materials such as silicon membranes and metal interconnects, which enable soft implantable bioelectronics. The most recent trend in implantable systems uses transient materials that naturally dissolve in body fluid after a programmed lifetime. Such biodegradable metallic materials are advantageous in the design of electronics due to their proven electrical properties. Collectively, this review delivers the development history of materials in implantable devices, while introducing new bioelectronics based on bioresorbable materials with multiple functionalities.

## 1. Introduction

In recent years, a variety of low-profile electronics have been developed for body implantable medical devices [1], such as the pacemaker, cardiac defibrillator, bladder stimulator, cochlear implants, and biosensors for the monitoring of pressure, flow, strain, and chemical sensors [2,3,4,5,6]. Such implanted systems are designed for long-term use in the human body, from a few months to several years. Therefore, a material’s biocompatibility is one of the most important features to be studied [7]. While there are many reviews and studies of strategies for investigating the foreign body reaction of the materials used in these devices, no systematic review has been reported for recent trends regarding the electronic materials used in medical devices. Considering the rapid development of implantable devices, such a review is critical to understanding both the types and major properties of the materials in order to investigate strategies for enhancing their functionality and biocompatibility. The primary purpose of implanted medical devices is either to diagnose physiological conditions in health, or stimulate organs for necessary functionality in the body. For diagnostics, electronics have been designed to offer accurate and real-time monitoring of important parameters via wireless telemetry systems. For organ actuation, electronic devices such as the pacemaker, cardiac defibrillator, balder stimulator, or artificial organs, have provided either mechanical or electrical stimulation to provide functions. In past decades, the aforementioned medical devices have used rigid and bulky electronic components to meet the required performance. However, these systems with high modulus materials develop unwanted inflammation or complications in the body, which bring critical health issues [8].

Recent advances in micro/nanofabrication technologies have enabled the miniaturization of functional electronic components to develop low-profile bioelectronics [9]. Specifically, various implantable sensors have shown dramatically reduced form factors, such that they can be implanted in the human body without disturbing fluid flow or causing tissue and bone loss. While the miniaturization significantly changed the trend of implantable medical devices, there remain significant hurdles caused by rigid materials. For example, a device implanted in joints or moving regions generates complications due to a mismatch between the material’s properties and the surrounding cells and tissues. In addition, minimally invasive surgeries cannot accommodate any rigid devices, since they are not deliverable with low-profile tools such as stents or catheters.

Here, new materials that offer mechanical compliance and flexibility can make great contributions for the next generation of implantable bioelectronics. Flexible materials such as functionalized polymers or thin-film metallic membranes have become a new frontier in body implantable bioelectronics, which has resulted in a significant increase in research outcomes. The most recent achievements include the finding of biodegradable metallic materials and their use in implantable systems with great electrical properties and functionalities. These devices can be programmed in a way that naturally dissolve in the body due to the controlled material’s density and inherent solubility in fluid. Even though many aspects of the materials and their systemic integration still need further study and investigation, the promising outcomes are very encouraging in the design of implantable bioelectronics that disappear after a certain lifetime without a second surgery. Overall, this review focuses on trends in the development of electronic materials that are being used in implantable bioelectronics. The various types of materials that are being used in medical devices and their key properties are summarized, and device functionalities and current challenges are discussed.

## 2. Traditional Materials Used in Implantable Bioelectronics

In past decades, a wide range of electronic materials has been used to develop implantable bioelectronics. Among them, silicon, silicon compounds, and stainless steel were popular due to the fully established fabrication process via high-throughput and high-yield micro/nanofabrication techniques. These materials work as functional parts in electronics, while medical devices incorporate other materials such as polymers and ceramics. Although these traditional materials are intrinsically rigid, advanced technologies in materials processing and manufacturing made them sufficiently flexible for implantable applications.

### 2.1. Examples of Traditional Electronic Materials

Table 1 includes a representative list of well-known, traditional materials that have been used in implantable biomedical systems, including silicon, glass (silicon dioxide, or quartz), ceramic (e.g., silicon nitride), and metal/metallic oxides. They are categorized as rigid materials, since they exhibit high Young’s moduli, high hardness, a high temperature processing limit, and low gas permeability. These materials are compatible with complementary metal–oxide–semiconductor (CMOS) processing, micro/nanofabrication, and integrated circuit design.

Silicon is commonly used as a structural material or bulk substrate in implantable medical devices, owing to its ease of micromachining with high resolution. Various MEMS fabrication techniques have been developed, including photolithography, etching, and deposition to enable the patterning on silicon layers over the decades, which can be used to fabricate different types of sensors for in vivo diagnostics. Typical pressure sensors that utilize a thin diaphragm suspended over the hard substrate are good example of the use of hard materials in implantable bioelectronics. Basically, the design of a silicon-based pressure sensor involves a vacuum sealed cavity enclosed on at least one side of the thin membrane, and the membrane deflects in response to the applied physiological pressure, which can be measured as capacitance changes or piezoelectric signals [29]. This pressure sensor design is robust, can be low-profile, can be integrated with planar inductor coils for wireless monitoring, and has been successfully applied in intraocular pressure sensing with a thin parylene coating [10,11,12,13,14,15] and cardiovascular monitoring [16,30,31]. Another example of a device would be a highly boron-doped silicon membrane, which has been used for the deflectable diaphragm on the silicon wafer developed by Chatzandroulis et al. [16]. Silicon itself is hard and stiff material, but it can be used as a thin membrane in implantable pressure sensors, enabling the dynamic measurement of arterial blood pressure fluctuations. Yoon et al. [17] developed a micro-telemetry pressure sensor, which was designed for intracranial pressure monitoring after the implantation of the shunt system. The patterned p+ silicon membrane functions as the diaphragm, and along with two Cr/Au electrodes, constitutes a capacitor in response to applied pressure. The capacitive pressure sensor was integrated with copper coils on a Pyrex glass substrate for wireless pressure monitoring.

**Figure 1:**
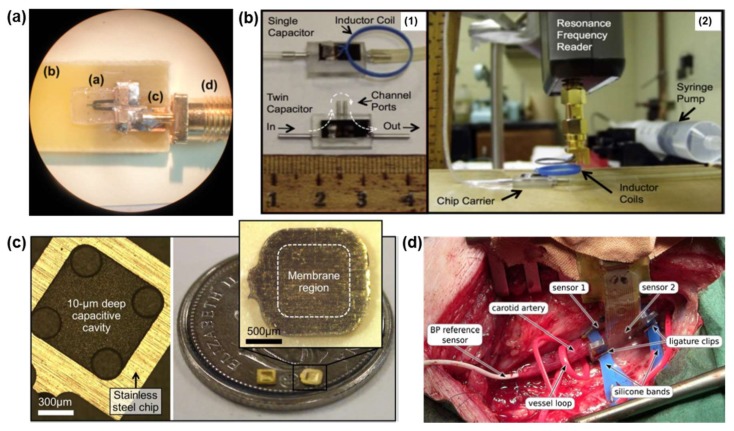
(**a**) Assembled blood pressure sensing device; (**a**) pressure sensor on, (**b**) FR-4 test board with, (**c**) transmission line, and (**d**) SubMiniature version A (SMA) connector. Reprinted with permission from Ref. [18], Copyright (2013), Springer Nature; (**b**) Flow sensors for cerebrospinal fluid sensing: (**1**) Test sensors with a single capacitor with inductor coil attached (**top**) and a twin-capacitor sensor without inductor coils (**bottom**); (**2**) Flow control unit (syringe pump) and spectrometer (resonance frequency reader) with a test sensor on a chip carrier. Reprinted with permission from Ref. [24], Copyright (2015), Elsevier; (**c**) Fabricated intravascular pressure sensors: (**left**) with a stainless-steel chip before membrane bonding and completed sensors; (**right**) a close-up of the diaphragm. Reprinted with permission from Ref. [27], Copyright (2014), Spring Nature; (**d**) Photograph of the optical blood pressure sensor units mounted onto the carotid artery of a domestic pig. The photo shows the operation site before measurement. Reprinted with permission from Ref. [35], Copyright (2012), Springer Nature.

Glass, mainly silicon oxide (SiO_2_) or single crystal quartz, is also a quite rigid material that is widely used in implantable wireless surface acoustic wave (SAW) sensors for blood pressure measurement [18,19,20]. Glass can be etched isotropically by hydrofluoric acid (HF), and anisotropically with deep reactive ion etching (DRIE) using SF_6_ or C_4_F_8_ [32,33] to create the desired patterns. The high-quality factor in resonators whose natural frequencies are insensitive to temperature variations around a desired operation point makes crystal quartz a suitable candidate for implantable devices. Metallic interdigital transducer electrodes were deposited on the piezoelectric quartz substrate, and a thin quartz membrane was bonded onto the substrate to create the diaphragm. The pressure sensor was supported by two rigid polyethylene terephthalate (PET) walls and by two silicone walls to improve the sensitivity (Figure 1a).

Silicon nitride (SiN_x_) is the chemical compound of the elements silicon and nitrogen; Si_3_N_4_ is the most thermally stable one. It is a ceramic material with a Young’s modulus of 300–320 GPa, a Poisson’s ratio of 0.27, a tensile strength of 350–400 MPa and a Vickers hardness of 13–16 GPa [21]. Si_3_N_4_ also has a non-toxic, biocompatible ceramic surface for functional human bone cells propagation in vitro, making it more attractive for orthopedic implants than other ceramic materials. In the implantable microelectromechanical (bioMEMS) sensor designed for orthopedic applications [22,23], the bioMEMS sensor is fabricated from a standard MEMS fabrication process, and Si_3_N_4_ is deposited using plasma-enhanced chemical vapor deposition (PECVD). Si_3_N_4_ was used as the dielectric layer sandwiched between two electrode layers due to its relatively high dielectric constant (~8) and low loss, resulting in a high Q-factor. The physical loading on the composite sensor layer results in the sensor’s capacitance change via the resonator’s structural deformation. In the implantable flow sensing design for cerebrospinal fluid flow monitoring [24,25,26], the capacitive sensor was fabricated from silicon wafers using the standard MEMS technologies, and silicon nitride was deposited using PECVD as the protection or insulation layer for the device. Low-stress silicon nitride (SiN_x_) was deposited on both sides of the wafer, and then on the top of the bottom electrode (Cr/Ni bi-layer) as the hard mask for the etching. The capacitive pressure sensor can detect the combined effect of cerebrospinal fluid (CSF) pressure, hydrostatic pressure, and flow by measuring the flexible membrane deflection. It has the sensitivity to measure clinically relevant ranges of slow-moving fluids such as the CSF in the brain, which is typically around 20 mL/h in a healthy individual (Figure 1b).

Metallic substrates, such as type −316 L stainless steel, were also used in some implantable devices. Chen et al. [27,28] developed the MEMS capacitive pressure sensor on a stainless steel chip, which is the same as the stent for integration with inductive stents by microwelding (Figure 1c). The use of stainless steel with a cavity as the substrate enabled a direct connection between the pressure senor and the stent, and thus eliminated the need for additional electrical leads at the joint, and improved the device reliability. The pressure sensor was fabricated from a gold–polyimide composite in order to create a sufficiently sensitive to the intravascular pressures with the capacitive electrodes and diaphragm structure. The whole device was packaged with parylene-C layers for electrical and chemical insulation from the fluid environment.

Some implantable medical devices utilized rigid circuits with sensing modules on the chip level to measure the corresponding physiological signals. These often consist of two units: a sensor unit to monitor the physiologically relevant signals such as blood pressure, and a control unit to perform A/D conversion, signal processing, and transmitting. In an implantable optical sensor for long-term blood pressure measurement [34,35], the correlation between pulse transit time and blood pressure was employed with light-emitting diodes (LEDs) and a silicon photodetector on a polyimide substrate. All of the substrates and sensors were encapsulated with silicone to achieve sensor conformability to the underlying tissues. The silicone strips allowed the attachment of the sensor unit directly onto arteries with a diameter greater than 4 mm without appreciable constriction, which added the flexibility in the measurement configurations, as shown in Figure 1d. An implantable accelerometer system [36] with the function of detecting the reflected wave transit time for blood pressure measurement was also developed by mounting digital accelerometers on the flexible polyimide foils. The microcontroller and radio frequency (RF) units on the chip form the rigid part in the sensing system, and connect with the sensor units by a meander structure. A parylene-C layer was deposited to offer protection from surrounding fluids and tissues.

### 2.2. Challenges and Limitations of Traditional Materials

There are big challenges regarding rigid and hard materials in bioelectronics, including biocompatibility and device delivery. The biocompatibility issue always occurs at the interface between implants and tissue or blood. The sensor materials should cause very little or no long-term damage to the artery and a long-term inflammation response from the surrounding tissues. As most of the materials mentioned above may not be biocompatible, they often require additional encapsulations (e.g., silicone, parylene) to shield the body from potential harmful materials to minimize the inflammatory response from the tissues and vessels. It remains challenging on the conformal adhesions between the encapsulation materials and the implantable electronic devices, which increases the fabrication complexity for those implantable medical devices [29,37,38]. As sharp edges and corners are present in the “hard” electronic device surface, it might impose serious damages to the relatively soft tissues during the surgical placement and implantation. In addition, most of the implantable medical devices fabricated from traditional “hard” materials are quite bulky, which could cause disturbance in the blood flow or even significant coagulation or clot after being implanted in the blood vessels.

Another critical issue in biomedical devices fabricated from traditional rigid materials is the mechanical property mismatch with surrounding tissues at the target site. Since human tissues and vessel walls are soft and stretchy, the high modulus materials in the device often cause excessive stress and multimodal deformation. For example, the microscale motion of an implanted device [39] caused severe damages to the tissues and exacerbated the foreign-body response of the immune system. The following consequences of the material’s mismatch include unexpected infection and tissue hardening [7,29]. To avoid such abnormalities, devices are required to have an additional surface encapsulation of soft, flexible materials, which minimizes the external stress to the implanted site.

The last challenging issue is related to the delivery of bioelectronics. The new trends of minimally invasive techniques in both vascular and orthopedic applications enable the device to be delivered with a tiny puncture either in the blood vessel, tissue, or bones. These techniques reduce the hospital stay of the patients, as well as reduce the risk and pain from open invasive surgical procedures. While these techniques are a new trend and are widely used for various implantable devices, rigid and bulky devices cannot be delivered via these methods. Any implantable bioelectronics created with rigid materials, if they are bulky and stiff, cannot be delivered by minimally invasive surgical techniques, losing the benefit for the patients. Therefore, new soft and flexible materials attract a lot of attention to accommodate these trends.

## 3. New Materials for Soft and Flexible Electronics

With the advancement of miniaturization fabrication techniques, micro/nanopatterning capability has been significantly improved. Additionally, new soft materials have been used for various implantable bioelectronics due to their unique and excellent properties, such as large elastic range, excellent biocompatibility, and easy fabrication. Soft material-based bioelectronics can be applied for various devices that require highly flexible properties, stretchable performance, and low-profile features maintaining the electronic function [40,41,42]. The flexibility of electronics is defined as whether their mechanical characteristics are bendable, foldable, or stretchable. In this section, two types (organic and inorganic) of soft materials are introduced.

### 3.1. Organic Materials

With the development of polymeric material fabrication technologies, a set of polymeric materials including polydimethylsiloxane (PDMS), medical grade silicone, parylene, polyimide, polyvinylidene fluoride-trifluoroethylene (PVDF-TrFE), and liquid crystal polymer (LCP) have been widely used in flexible and soft electronics as substrates, sensing components, and encapsulations. Most of the polymeric materials are soft, lightweight, RF-transparent and low cost, and hence can address the current challenges associated with metallic and ceramic materials for implantable electronics. Table 2 summarizes the list of applications of several important polymeric materials in flexible electronics.

**Figure 2:**
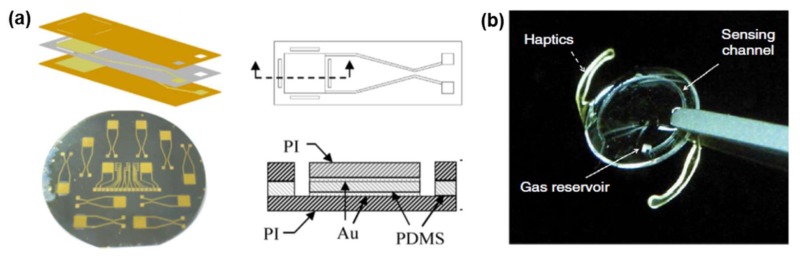

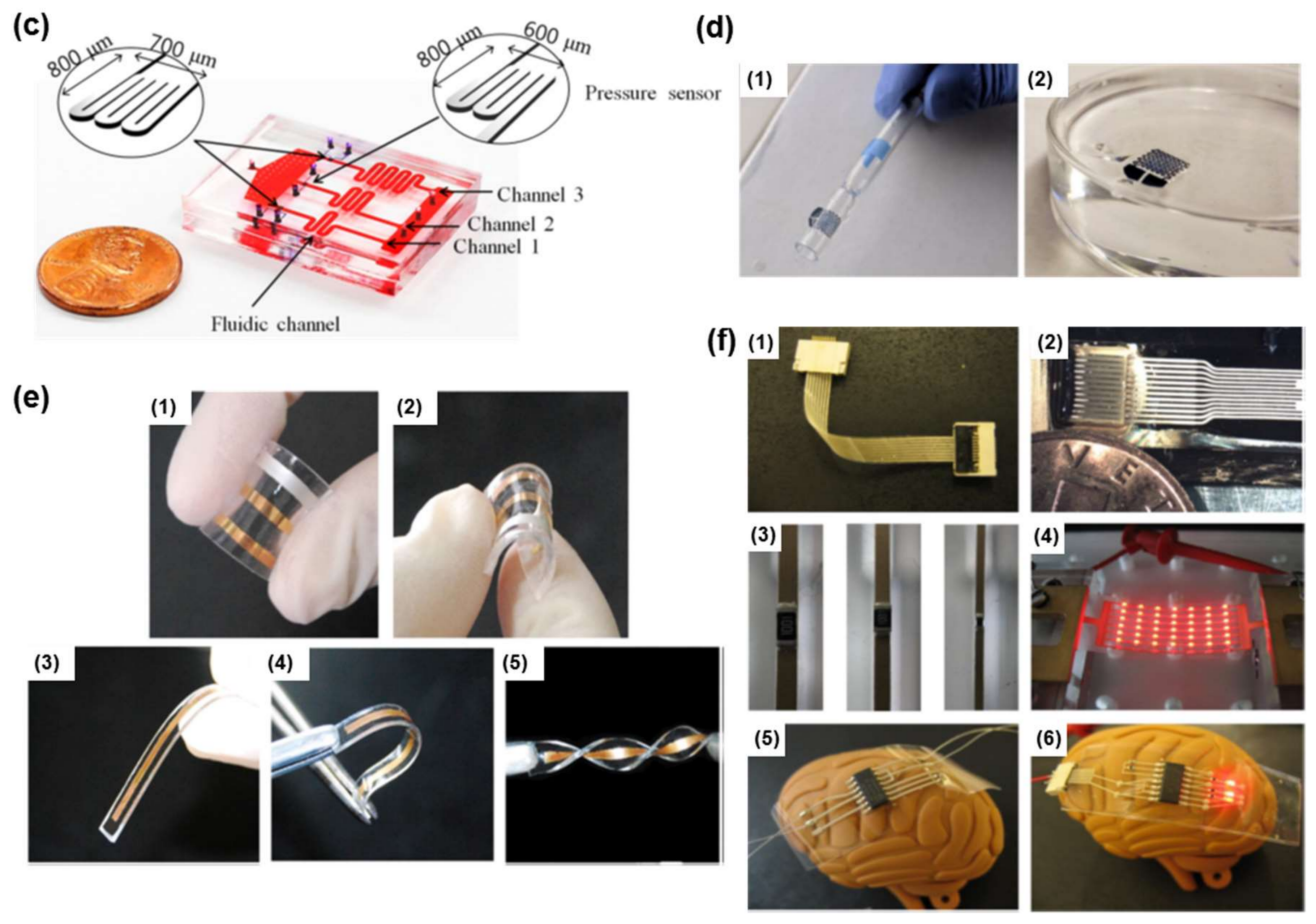
(**a**) Structure of the flexible capacitive pressure sensor. Reprinted with permission from Ref. [46], Copyright (2007), Elsevier; (**b**) Photograph of the microfluidic pressure sensor embedded within the intraocular lens. Reprinted with permission from Ref. [43], Copyright (2014), Springer Nature; (**c**) The intraocular pressure (IOP) sensor integrated in the microfluidic device consists of three PDMS layers: sensor, thin-film, and fluidic-channel layers. Reprinted with permission from Ref. [44], Copyright (2015), MDPI AG; (**d**) A flexible biosensor fabricated on the PDMS film showing the pattern flexibility (left, **1**) and water stability (right, **2**). Reprinted with permission from Ref. [86], Copyright (2018), Elsevier; (**e**) Pictures of a flexible PDMS-based three-electrode sensor; Top view **1** and side view **2** of the three-electrode sensor, straight **3**, bent **4** and twisted **5** working electrode. Reprinted with permission from Ref. [49], Copyright (2010), Elsevier; (**f**) Fabrication of soft electrical circuits on PDMS integrating commercially available electrical components. **1** Picture of a ribbon cable with eight conductors clamped on both ends by a commercial zero insertion force (ZIF) connector; **2** Picture of a custom-made miniature connector with 12 contacts; **3** Pictures of chip resistors bonded on conductive tracks (from left to right: 0805, 0603 and 0402 packages); **4** Picture of a large array of surface mounted device (SMD) light-emitting diodes (LEDs) bonded on a soft printed circuit board (PCB); **5** Picture of a 2-kHz clock generator produced on a 0.2-mm thick soft single-sided PCB that conforms to a plastic brain. **6** Picture of a 1-Hz clock generator with LEDs to display the output levels produced on a 0.7-mm thick double-sided soft PCB. Reprinted with permission from Ref. [84], Copyright (2014), Springer Nature.

PDMS, a type of silicone elastomer, offers distinct advantages over other materials in implantable pressure-sensing applications, such as (1) a unique flexibility due to a low Young’s modulus (<100 MPa); (2) imperviousness to fluids; (3) high dielectric strength (~14 V·µm^−1^); (4) low chemical reactivity; and (5) proven biocompatibility [81,82]. Therefore, PDMS is selected as the dielectric layer in capacitive pressure sensors. It was reported to be used in highly sensitive capacitive sensors designed for pressure and oxygen content measurement within the heart and blood vessels [45], and interface pressure measurement between the nerve trunk and cuff electrode for nerve tissue health monitoring (Figure 2a) [46]. PDMS polymer is also favorable for fabricating microfluidic channels in bioelectronic devices due to its optical transparency, flexibility, and suitability for soft lithography. The biocompatibility of PDMS also suggests that it might ultimately be possible to embed microfluidic devices in vivo for biomedical analysis [83]. Araci et al. [43] developed a novel passive intraocular pressure (IOP) sensor implant for glaucoma diagnosis and monitoring using standard soft lithography out of PDMS (Figure 2b). Jung et al. [44] fabricated the fluidic channel from PDMS in a resistive-type pressure sensor embedded in a microfluidic system (Figure 2c). Also, PDMS can function as a flexible substrate for soft and ultra-compliant electronic devices and encapsulation materials to mechanically and chemically decouple devices from their environment. Patterned structures of stretchable and electrically conductive materials (e.g., gold electrodes) were embedded in a thin PDMS substrate (Figure 2d–f) [47,48,49,84,85,86] to allow for high conformability over any soft/curvilinear surfaces in vivo, thereby enabling a broad range of non-invasive or minimally invasive and implantable systems to address clinical needs [87]. The PDMS-based devices are flexible enough without inducing irreversible deformations or fatigue after the devices were bended, twisted, rolled, or stretched (Figure 2e).

**Figure 3:**
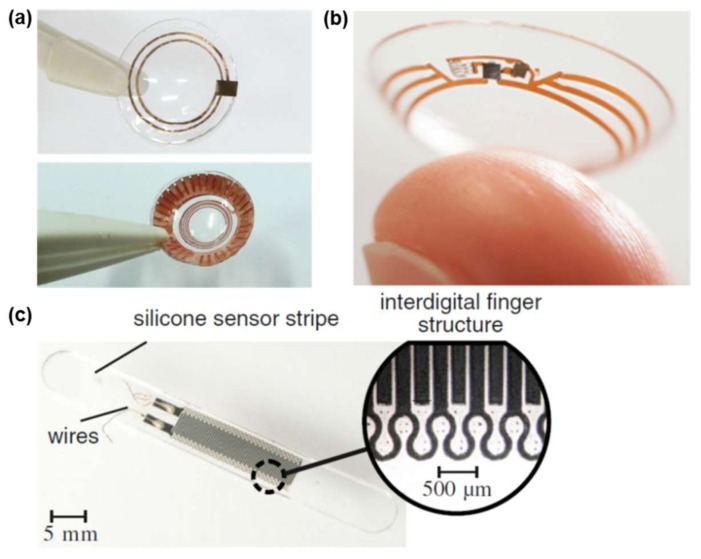
(**a**) A contact lens with sensing elements embedded in a silicone rubber. Reprinted with permission from Ref. [52,53], Copyright (2013, 2014), Elsevier; (**b**) The contact lens sensor under co-development by Google and Novartis. It measures glucose concentration in tears using a miniaturized electrochemical sensor embedded into a hydrogel matrix, Reprinted with permission from Ref. [54], Copyright (2014), John Wiley and Sons; (**c**) Silicone sensor strip with the dimensions of 40 mm × 5 mm × 0.5 mm. Reprinted with permission from Ref. [51], Copyright (2012), Springer Nature.

Medical grade silicone, another type of silicone elastomer, is Food and Drug Administration (FDA)-approved for biocompatibility to be used in biomedical implants. With exceptional properties such as high tear strength and outstanding elasticity over a wide temperature range, it also exhibits a tensile strength of <10 MPa with the elongation of 300–1000% [88,89], which adds more flexibility to the silicone-based medical devices compared with PDMS. As the strong Si–O–Si (siloxane) backbone provides enhanced chemical inertness and exceptional flexibility, medical grade silicone is considered an ideal candidate for the substrate materials in implantable medical devices with ultracompliance. The important optical properties of the substrate for contact lens sensors are transparency and a sufficiently high refractive index. Therefore, soft contact lens sensors were fabricated by embedding resonance circuits in medical grade silicone layers (NuSil) for continuous IOP monitoring [52,53,54]. The ultracompliance of the silicone enabled IOP monitoring from the curvature of the cornea (Figure 3a,b). Aqulina et al. [50] fabricated the intracranial pressure (ICP) monitor with the flexible printed circuit board (PCB) coated with medical grade silicone rubber. Medical grade silicone was also used in a capacitive strain gauge housing strip, which can be wrapped around the artery to monitor the blood pressure changes by measuring the blood vessel deformation (Figure 3c) [51]. The Young’s modulus of the device is comparable to that of the blood vessel, thus offering the flexibility to minimize the blood flow disturbance.

**Figure 4:**
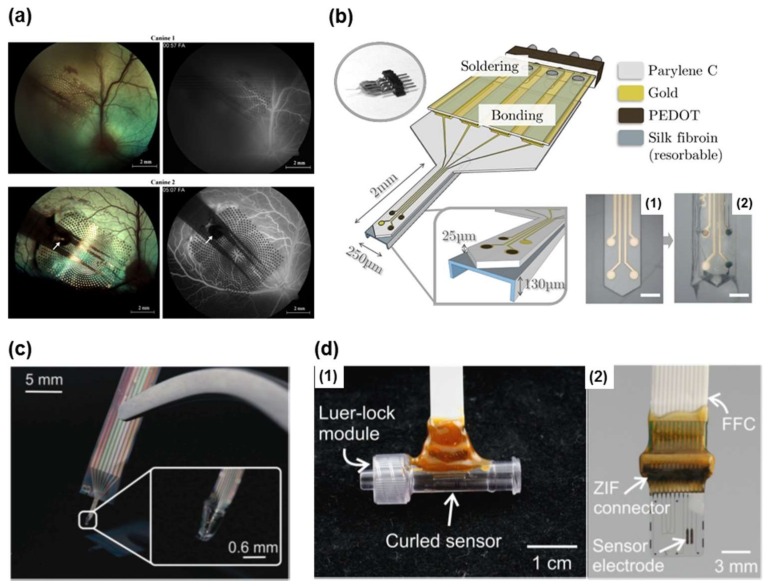
(**a**) Fundus photographs (**left**) showing parylene multi-electrode arrays (MEAs) tacked to the right retina of both animals, and fluorescein angiographies (FAs) (**right**) showing normal vessel perfusion under the arrays. Reprinted with permission from Ref. [62], Copyright (2008), Elsevier; (**b**) Schematic representation of a parylene C-based neural probe with three poly (3,4-ethylenedioxythiophene) (PEDOT)-nanostructured electrodes (40 µm in diameter), and one gold electrode as control. Two photographs show the probe tip **1** before and **2** after PEDOT nano-structuration and silk integration (scale bar 150 µm). Reprinted with permission from Ref. [91], Copyright (2017), Elsevier; (**c**) Fabricated flexible parylene sheath neural probe with integrated parylene cable. Reprinted with permission from Ref. [61], Copyright (2012), Royal Society of Chemistry; (**d**) **1** Packaged patency sensors in inline modules for benchtop testing; **2** Electrically packaged parylene device with final electrode design using a ZIF connector and integrated flat flexible cable (FFC). Reprinted with permission from Ref. [59], Copyright (2016), Springer Nature.

Parylene C, or poly(dichloro-*p*-xylylene), is a polymeric material that is widely used as a substrate or encapsulation material for biomedical devices due to its FDA-approved biocompatibility, chemical and biological inertness, good barrier properties with low water permeability and absorption, and its functionality as an electrical insulator. The Young’s modulus is 1–4 GPa, the tensile strength is 40–110 MPa, and the elongation at break is 7.5–42% [90]. The tensile strength is sensitive to thermal treatment such as annealing, as well as deposition pressure due to the structural crystallization. Parylene-C layers shows a good adhesion to underlying materials such as Si_3_N_4_, platinum, and itself with an adhesion promotor, Silane A-174 (methacryloxypropyltrimethoxysilane) [90]. Parylene C was selected as the diaphragm and disk substrate for the continuous IOP monitoring of glaucoma patients. The thin parylene-C membrane is sensitive to the applied pressure, and capacitance changes can be induced by membrane deformation. The whole IOP sensor was also packaged and sealed by a thin parylene-C layer to ensure the biocompatibility in the intraocular environment for in vivo tests [55,56,57,58]. Parylene C is also a popular flexible substrate material for neural signal recording applications. In high surface-area electrode arrays for high-density simulation and recording in retinal and spinal cord prosthetics, the thin-film platinum and iridium electrodes are embedded in a flexible parylene-C substrate (Figure 4a) [62]. The three-dimensional (3D) sheath neural electrode probes for neural recordings are also constructed on the flexible parylene-C substrate (Figure 4b,c) [60,61,64,91]. The 3D sheath probe arrays were formed by thermal molding of the surface micromachined parylene-C channel, which allows for the recordings on large areas and multiple sites of interest. In a novel microbubble pressure sensor design, a pair of platinum electrodes were embedded in a parylene-C substrate for hydrocephalus treatment monitoring [63]. This unique sensing mechanism utilized the electrochemical impedance measurements of electrolytically generated microbubbles in contact with the parylene-C surface. Another example of a parylene-C based device is the patency sensor for proximal hydrocephalus shunt occlusion detection [59]. Platinum electrodes were patterned on the parylene-C substrate to measure the electrochemical impedance with respect to cerebrospinal fluid flow (Figure 4d). This inline module can be implanted into shunt to enable quantitative and accurate monitoring of shunt performance.

**Figure 5:**
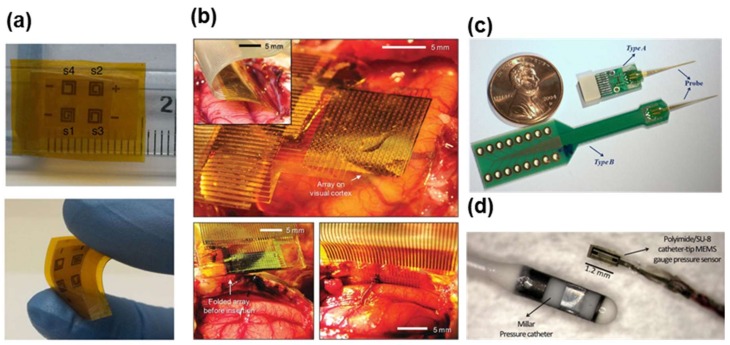
(**a**) Pressure mapping with a 2 × 2 flexible array of 2 × 2 mm^2^ sensors on Kapton film showing the size (**top**) and flexibility when bent (**bottom**). Reprinted with permission from Ref. [65], Copyright (2014), Springer Nature; (**b**) Animal experiment using feline model. (**Top**) A flexible, high-density active electrode array was placed on the visual cortex. Inset, the same electrode array was inserted into the interhemispheric fissure; (**Bottom left**) Folded electrode array before insertion into the interhemispheric fissure; (**Bottom right**) Flat electrode array inserted into the interhemispheric fissure. Reprinted with permission from Ref. [69], Copyright (2011), Springer Nature; (**c**) The National Chiao Tung University (NCTU) probe was bonded onto two different types of PCB. *Type A* assembly was used for chronic recording, and *Type B* was used for acute recording in free-moving animals. Reprinted with permission from Ref. [66], Copyright (2009), Elsevier; (**d**) Assembled polyimide/SU-8 catheter-tip microelectromechanical (MEMS) gauge pressure sensor in comparison with a commercial Millar Mikro-Cath™ disposable pressure catheter. Reprinted with permission from Ref. [94], Copyright (2012), Springer Nature.

Polyimide (PI) is a polymer of imide monomers; it exhibits a Young’s modulus of 1.5–3 GPa and a tensile strength of 70–100 MPa. The elongation at breakage ranges from 2% to 15%, depending on the chemical structure [92]. Polyimides show high heat resistances and high glass transition temperatures, and are stable up to a temperature of 440 °C [92]. Polyimides are widely used in electronic devices as passivation or insulation materials and substrate layers because of their excellent thermal and chemical stabilities, low dissipation factors, and low dielectric constants. Polyimide film (Kapton tape) was used as the insulation layer for copper coil patterns to form an inductor in a minimally invasive pressure sensor for continuous IOP monitoring [15]. Chen et al. [65] developed the wireless pressure monitoring and mapping system with ultrasmall sensor patterns on the flexible polyimide substrate layer (Figure 5a). Viventi et al. [69] fabricated the ultra-thin and flexible electrode arrays on a 12.5-µm thick polyimide film to record the spatial properties of brain activity in vivo (Figure 5b). The extreme flexibility of the device is achieved by reducing the array and substrate thickness to minimize the induced strain during the folding. The extreme flexibility of the device enabled the access to rarely explored cortical areas for neural activity mapping. The highly flexible polyimide substrates for electrode arrays allow for the conformal coverage to the tissues, and cause less harm to the implant site, which makes them suitable for chronic neural activity recordings (Figure 5c) [66,67]. However, the polymer may experience buckling caused by the insertion force during the device implantation [68]. Polyimides are also used in structural components in medical sensors. In a wireless IOP sensor, the copper inductor pattern was deposited on top of the flexible polyimide membrane while a high frequency NiZn ferrite was attached on the bottom [70]. The inductance was varied, with a variable distance between the ferrite material and the inductor pattern with respect to the applied pressure. Shin et al. [71] developed a dual-mode IOP sensor with two separate diaphragms (flexible polyimide and elastomer membrane) to conduct the changes in inductance and capacitance in order to enhance the device performance. A thin polyimide film was also used as the diaphragm in the capacitive pressure transducer for implantable cardiovascular applications (Figure 5d) [93,94].

Polyvinylidene fluoride (PVDF) and its copolymers polyvinylidene fluoride trifluoroethylene (PVDF-TrFE) are attractive in a broad range of applications, including acoustic transducers and electromechanical actuators, because of their piezoelectric response (generating electrical signals while it is mechanically deformed) and thermal and chemical stability. PVDF polymer exhibits a relatively high room-temperature dielectric constant (>40) and a high electrostriction (strain >4%). Also, it is a thin, flexible, lightweight material and can sustain higher strains (40–140% elongation) compared with other ferroelectric materials [95,96]. PVDF-TrFE piezoelectric films were used to fabricate the flexible diaphragm in a dual-mode intracranial pressure sensor. The PVDF-TrFE diaphragm can operate in a capacitive and resonant mode, allowing for high linearity over small pressure changes with insensitivity to environmental temperature variations and high sensitivity with easy adaption for wireless applications [73]. A PVDF-TrFE copolymer film pressure sensor can also be integrated with a catheter for intravascular measurements. PVDF-TrFE copolymer was spin-coated into thin films (1-µm thick) to tap the near β-phase formation, and it showed no electrical pooling or mechanical stretching. A PVDF-TrFE film can be then patterned using a standard lithography process, and fabricated pressure sensors can be easily mounted on catheter surfaces for real-time measurements [74,75]. Another application of PVDF polymer is simultaneous heartbeat and respiration monitoring based on the piezoelectric response to pulsatile vibrations and periodical deformations on the chest wall [72].

Liquid crystal polymer (LCP) belongs to the family of aromatic polymers. It exhibits a Young’s modulus of 2–10 GPa, a tensile strength of 270–500 MPa, and a relatively low dielectric constant (~2.9 at 1 MHz). The uniqueness of the LCP material is its much lower moisture absorption rate (<0.04%) compared with polyimide and parylene-C materials [77,97]. As the moisture-generated surface may cause reliability issues for an electrical circuit and its components, its application in LCP allows for possibly longer-term device implantation. LCP materials have been used for substrates and encapsulations in bioelectronics due to their superior characteristics in heat resistance, chemical stability, mechanical flexibility, and biocompatibility. LCP was used as a soft and flexible host substrate for a miniature capacitive pressure sensor in IOP fluctuation monitoring [58]. The patterning of LCP films is compatible with conventional silicon-based MEMS processing, and can be easily integrated with the capacitive sensing components. In brain and vagal stimulation applications, an iridium oxide electrode was deposited on LCP as a flexible substrate for long-term electrical stimulation [79]. Lee et al. [78] developed an implantable light-emitting phosphor-coated GaN light-emitting diode (LED) on an LCP substrate for prostate-specific antigen detection. Jeong et al. [76,77] fabricated a retina prosthetic implants on the LCP film substrate whose structure is conformable to the eye surface; it allows for the attachment of the whole implant to the eyeball surface. Also, the LCP encapsulation provides long-term reliability without electrical degradation. LCP is also selected as the packaging materials for an implantable active IOP monitoring system consisting of a MEMS pressure sensor, a power storage array, an application-specific integrated circuit (ASIC) for signal processing, and a monopole antenna. The in vivo studies showed the least amount of fibrous encapsulation and inflammation on LCP-packaged devices [80].

### 3.2. Inorganic Materials

Flexible and stretchable electronics have the capabilities to absorb high levels of strain without fracture or performance degradation. As most inorganic materials are intrinsically brittle, the strategy for achieving flexibility and stretchability is to combine the bendable designs with layouts that enable the device’s out-of-plane motion. One strategy of configurations in flexibility for inorganic materials involves the construction of inorganic materials embedded in elastomer substrates, with significant applied strains absorbed by the elastomer substrates. These elastomer substrate materials have been reviewed in the previous section. Another strategy exploits coiled-spring or S-shaped (i.e., serpentine) interconnect structures to accommodate the applied strains [98]. In this section, we are going to review the inorganic materials that achieve high applied strains through serpentine interconnect layouts.

Transferrable monocrystalline silicon nanomembrane (Si NM) is a suitable candidate active material for fabricating fast flexible electronics due to its material uniformity, low interfacial stresses in bonded configurations, mechanical flexibility and durability, equivalent electrical properties to the bulk silicon, and ease of processing at a low cost [99,100,101]. Therefore, flexible or even stretchable devices can be designed using Si NMs. The manufacturing of Si NMs remains challenging due to their ultra-low profile and associated frangibility. Recently, transfer printing technology has been developed to retrieve the Si NM designs from the source wafers. Thus, silicon NMs can be patterned using photolithography and reactive ion etching (RIE), then released from SOI substrates completely and transferred to a versatile thin and flexible substrate with microscale precision using transfer printing techniques. With the integration of an ultraflexible substrate, the sensors fabricated from Si NMs can accommodate the extreme bending into a small radius of curvature, or even folding, without mechanical failures. An application of emerging nanomembrane technologies involves the intimation coupling of flexible or stretchable electronics with biological tissues such as heart and brain [102]. An example of bio-integrated electronic devices is a conformal bio-interfaced sensor system consisting of Si NM transistors configured to map cardiac electrical activity directly in vivo [103]. Doped silicon NMs were patterned from silicon wafer before being transfer printed onto the polyimide substrate. The whole system was insulated from the wet environment using a multilayer barrier strategy. With the applications of Si NMs, this device combined the high-performance transistors with medium-scale levels of integration to create the electrodes on a flexible substrate, which enabled the adhesion to the constantly moving epicardial surface without penetrating the tissues. Si NM transistors were also used as electrode arrays for direct brain activity mapping [69]. High-density electrode arrays allow for high spatial resolution over a larger region of brain with an improved signal-to-noise ratio.

Metallic materials such as gold and copper are rigid; however, they can be formed into wavy shaped interconnects to achieve stretchability. Deformations primarily occur at the curved edges of the serpentine interconnects to accommodate applied strains, while rigid active device regions still remain non-stretched. By optimization the thickness, shape, and curvature of the interconnects, the device can be stretched up to 70% without significantly affecting the electrical properties [98]. These stretchable electrode designs were demonstrated in the various applications such as stretchable supercapacitors, transistors, LED displays and touch-panel displays, wearable electronic devices, artificial skins, and muscles [104]. However, only a few implantable medical devices for healthcare monitoring were reported. An example of metallic interconnects in flexible bioelectronics is a miniaturized pH sensor array embedded in an elastomer substrate to achieve high surface conformal monitoring of the beating heart undergoing ischemia in a minimally invasive fashion [105]. The gold traces defined the sensing electrodes and contact pads, and a thin bilayer of Cr/Au was patterned to define the serpentine structure as interconnects to minimize the material strains. This pH sensor array can be either integrated with inflatable balloon catheters or have direct contact with an endocardial surface.

## 4. Biodegradable Materials for Transient Electronics

Biodegradable materials are a category of materials that can be degraded in vivo, either enzymatically or non-enzymatically, to produce biocompatible or toxicologically safe byproducts that can be eliminated by the normal metabolic pathways. Therefore, the biodegradability of transient electronics involves the breakdown of materials, which is mediated chemically and biologically into smaller fragments that can be dissolved or absorbed by the body [106].

### 4.1. Metallic Biodegradable Materials

Table 3 summarizes the common biodegradable metallic materials (Mg, Zn, and Fe) that are used as electrodes and interconnects in transient electronics. Conventional metallic materials in transient electronics are appealing because of their low electrical resistance, stable properties, and biocompatibility. With the exception of tungsten (W), all other metallic elements are essential for biological functions. The degradation behaviors of these metallic materials can be affected by various factors, and the electrical dissolution rates in thin film forms can be different compared with corresponding bulk materials [107]. Magnesium and magnesium alloys have been considered as potential candidate materials for short-term implants due to their high reactivity in corrosive media environments such as biofluids. As magnesium is present in large amounts in human bodies, the biodegraded magnesium materials can be absorbed. The key issue for the magnesium is the need to reduce the degradation rate in the human fluid environment for transient electronics applications, and the release of the degraded products should be within the human absorption level. It has been found that the degradation rate of magnesium can be reduced by magnesium purification, selective alloying, and anodized coating [108,109]. An example of implantable transient electronic devices that can provide thermal therapy to control surgical site infections uses magnesium as the conductors, magnesium oxide for the dielectrics, silicon nanomembrane (Si NMs) as the semiconductors, and silk for the substrate and encapsulation. After a time scale of 15 days, the device disappeared, with only remnants of silk left [110]. In another example, iron and zinc bilayers were used as the sensor conductor materials in combination with biodegradable polymers as insulation and packaging materials for a wireless pressure sensor [111]. The Zn layer allows for the formation of high-Q elements for conductors. Due to the slow degradation rate of pure Zn in a saline environment, an electrical bilayer on the biodegradable poly-l-lactide (PLLA) and polycaprolactone (PCL) layer was formed with the combination of Fe, resulting in a rapid and controllable degradation. The in vitro degradation tests revealed that the weight loss rate for the Zn/Fe bilayer in saline was 0.46 mg/(cm^2^·h), which is tenfold higher than that of pure Zn. Therefore, the degradation rate of the Zn/Fe bilayer can be tailored through modulation of the Zn to Fe exposed surface ratio. Metallic foils such as Fe, Mo, Zn, and Mg can also be used as the substrates for transient *n*-channel metal oxide semiconductor field effect transistors (MOSFETs) [112]. In contrast to the polymer substrates, which can swell and crack the supported electronic structures in biofluids, thin film metallic foil substrates are more robust, compatible for direct device fabrication, and thermally stable to provide hermetic protection. The dissolution behaviors for degradable metals (Fe, Mo, Zn, and Mg) are affected by the thickness, grain structure, and surface morphology in the form of thin film foils. These biodegradable metallic foils can be used in transient electronics for temporary biomedical implants and monitors, with a performance comparable to conventional non-transient materials.

### 4.2. Polymeric Biodegradable Materials

Biodegradable polymeric materials have been attractive in fabricating bioresorbable devices over the recent decades. These categories of materials can be degraded in vivo without the need for device retrieving after implantation. Also, most of them are soft and flexible, which can be used in stretchable and flexible medical devices.

**Figure 6:**
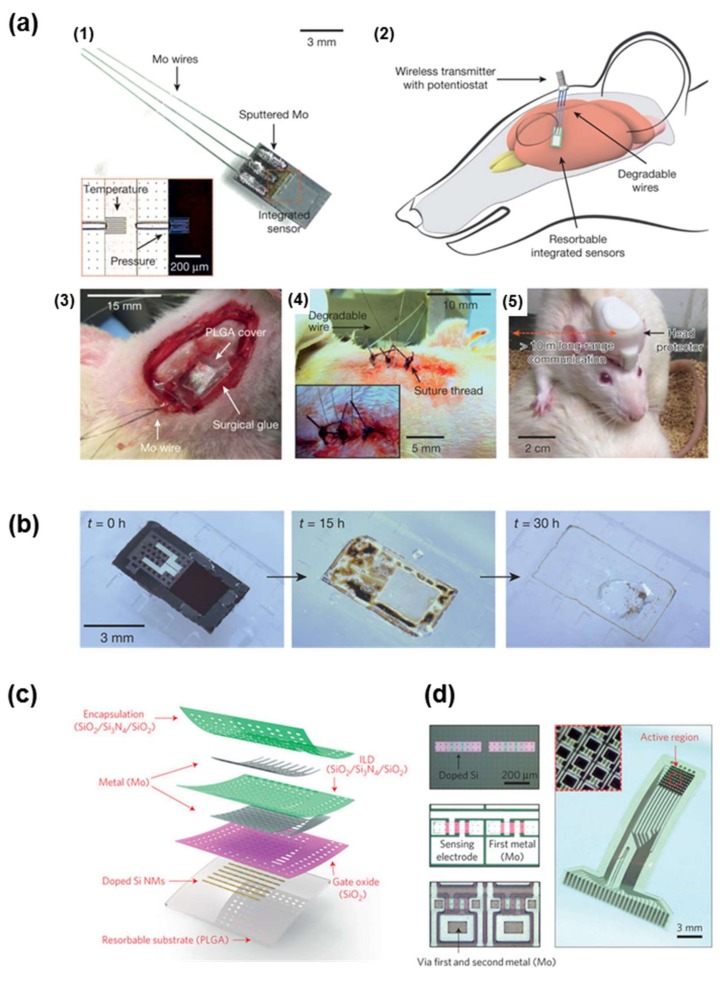

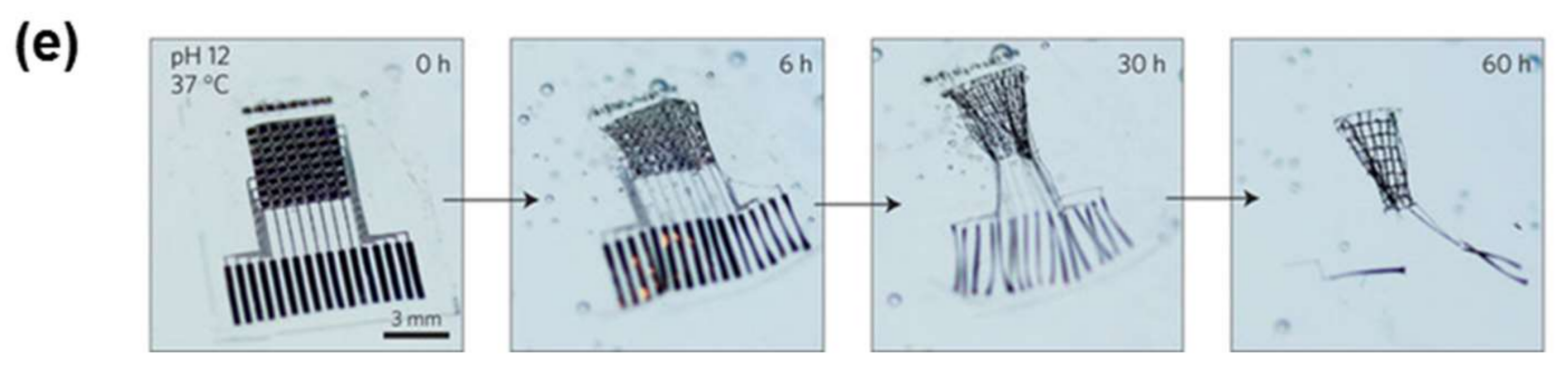
(**a**) Bioresorbable interfaces between intracranial sensors and external wireless data-communication modules with percutaneous wiring. (**1**) Image of bioresorbable pressure and temperature sensors integrated with dissolvable metal interconnects; (**2**) Diagram of a bioresorbable sensor system in the intracranial space of a rat; (**3**) Demonstrations of an implanted bioresorbable sensor in a rat and (**4**) sutured individual; (**5**) Healthy, freely moving rat equipped with a complete, biodegradable wireless intracranial sensor system. Reprinted with permission from Ref. [114], Copyright (2016), Springer Nature; (**b**) Images collected at several stages of accelerated dissolution of a bioresorbable pressure sensor upon insertion into an aqueous buffer solution (pH 12). Reprinted with permission from Ref. [114], Copyright (2016), Springer Nature; (**c**) Bioresorbable, actively multiplexed neural electrode array in an exploded-view rendering. Reprinted with permission from Ref. [115], Copyright (2016), Springer Nature; (**d**) Optical micrograph images of a pair of unit cells at various stages of fabrication (**left**) and a picture of a complete system (**right**). Reprinted with permission from Ref. [115], Copyright (2016), Springer Nature; (**e**) Images collected at several stages of accelerated dissolution of a system immersed into an aqueous buffer solution (pH 12) at 37 °C. Reprinted with permission from Ref. [115], Copyright (2016), Springer Nature.

Polylactic-*co*-Glycolic Acid (PLGA) is a copolymer of polylatic acid (PLA) and polyglycolic acid (PGA), and can dissolve in a wide range of common solvents, including chlorinated solvents, tetrahydrofuran, acetone, or ethyl acetate. In water, PLGA biodegrades by hydrolysis of its ester linkages. The degradation rate of PLGA is dependent on different factors, including the initial molecular weight of the monomers, the chemical composition, and the exposure time to fluid. The degradation time varies from 1–2 weeks to 5–6 weeks depending on the lactic acid (LA): glycolic acid (GA) ratio. Also, PLGA exhibits the Young’s modulus of 2 GPa with the elongation of 3–10%. It can be formed into a variety of sizes and shapes such as films, porous scaffolds, hydrogels, or microspheres. Therefore, PLGA is considered the best defined biomaterial available as a drug delivery carrier due to its design and controlled biodegradation rate [120,121]. Kang et al. [114] fabricated a silicon-based piezoresistive wireless sensing system onto a 30-µm thick PLGA substrate for intracranial pressure and temperature monitoring during traumatic brain injury (Figure 6a). The uniqueness of the sensing device is its ability to dissolve completely into biocompatible byproducts when immersed in aqueous solutions such as cerebrospinal fluid (CSF) (Figure 6b). A thin, flexible electrode array based on monocrystalline silicon (Si NM) structures with an insulation layer of SiO_2_ was constructed on a substrate of PLGA as a platform for the high-speed spatiotemporal mapping of brain activities (Figure 6c,d) [115]. The flexibility of the device allows for the conformal contact and chronically stable interfaces with neural tissues. Also, the whole device and materials are estimated to dissolve in biofluids completely in two months (Figure 6e). A transient hydration sensing system consisting of phosphorous-doped silicon for electrodes, magnesium for contacts/interconnects, and silicon dioxide for the dielectric layer, was fabricated on a PLGA substrate designed for wound-healing process monitoring [122]. The whole device will disappear over several months, as PLGA is the last to dissolve among all of the materials in this device. The challenging issue is the swelling of substrate material such as PLGA, which will lead to fracture/disintegration of the supported device structure during the dissolution. Another group reported the use of poly-l-lactide (PLLA) polymer, which is a variant of PLGA, as a piezoelectric force sensor designed for intra-organ pressure monitoring by employing the piezoelectric behaviors of the material [113]. The sensor includes two piezoelectric PLLA layers, sandwiched between a molybdenum or magnesium electrode and a polylactic acid (PLA) encapsulation layer. This piezoelectric force sensor completely degrades and breaks down after about two months.

**Figure 7:**
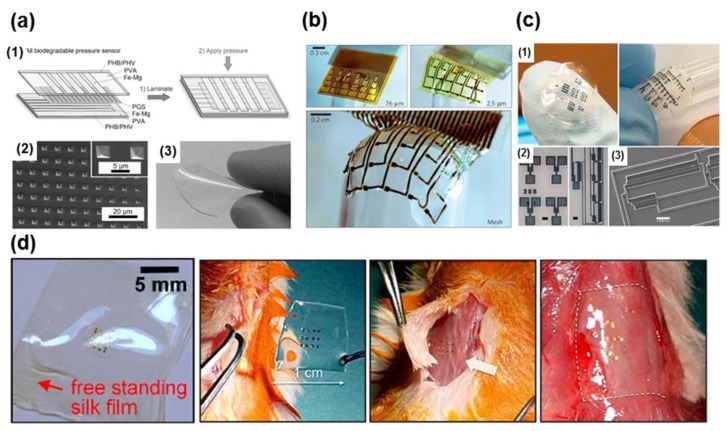
(**a**) Schematic design and fabrication of a fully biodegradable and flexible pressure sensor array from microstructured poly(glycerol sebacate) PGS films. (**1**) Schematic of the final fabrication step of the fully biodegradable pressure sensor and its device structure; (**2**) SEM image of the microstructured PGS film. Two-dimensional (2D) array of square pyramids are formed into PGS from a PDMS mold; (**3**) PGS biodegradable elastomer film. Reprinted with permission from Ref. [124], Copyright (2015), John Wiley and Sons; (**b**) Images of electrode arrays (76 μm sheet in left top, 2.5 μm sheet in right top and 2.5 μm mesh in bottom panel) wrapped onto a glass hemisphere. Reprinted with permission from Ref. [126], Copyright (2010), Springer Nature; (**c**) Formation of complex microstructures via photolithography. (**1**) Large area micropatterns of PEDOT:PSS can be formed on flexible and conformable silk fibroin sheets, (**2**) Optical micrographs and (**3**) SEM images of PEDOT:PSS micropatterns on glass. Scale bars = 100 µm. Reprinted with permission from Ref. [118], Copyright (2016), Elsevier; (**d**) Ultra-thin devices on a flexible silk substrate and the results of the animal toxicity test: image before (second image), shortly after (third image), and two weeks after (fourth image) implantation. Reprinted with permission from Ref. [117], Copyright (2009), AIP Publishing LLC.

Wang et al. [123] first reported the development of a new biodegradable polymer with improved mechanical properties and biocompatibility, poly(glycerol-sebacate) (PGS). PGS exhibits a Young’s modulus of 0.05–2 MPa, the tensile Young’s modulus of 0.28 MPa, and elongation at breakage is larger than 260%. PGS degrades by 17% after 60 days in phosphate-buffered saline (PBS) solution, and the degradation rate can be tailored. A pressure sensor array designed for cardiovascular monitoring was developed by sandwiching a PGS elastic dielectric layer between two iron–magnesium electrode layers (Figure 7a) [116,124]. The pressure response stays stable, even at a bending radius down to 27 mm. The key element in this design is the PGS layer, which retains the device performance even after prolonged exposure to the degrading environment. The device is expected to degrade completely after a few months. Lewitus et al. [125] also synthesized a new biodegradable polymer from polyethylene glycol (PEG) and desaminotyrosyl-tyrosine (DT) to enhance the degradation as the carriers for neural probe applications.

Silk is another appealing material as a temporary and soluble supporting substrate for transient electronics. Silk can be obtained from the cocoons of the larvae of the silkworm, and it offers the advantages over other biodegradable materials, including optical transparency, mechanically flexibility in thin-film form, compatibility with aqueous processing, biocompatibility, and bioresorbability with a controlled degradation rate. A silk fibroin solution has the ability to crystallize through protein self-assembly with exposure to the air to yield a class of patterned freestanding films or a mechanically robust substrate for biodegradable devices with the ability to control thickness [119]. Kim et al. [117,126] integrated electrode arrays designed for passive neural recording on the silk fibroin substrate (Figure 7b,c). It allows for the spontaneous, conformal wrapping on the curvilinear surfaces of brain tissues once the silk was dissolved in the biofluids. Also, the ultra-thin structure minimizes the stress on the tissue while ensuring highly conformal coverage. The dissolution rate of silk can be programmed with ethanol treatment in neural mapping applications. An electrochemical biosensor designed for dopamine and ascorbic acid detection was also developed by constructing conductive Poly(3,4-ethylenedioxythiophene): poly(styrene sulfonate) (PEDOT: PSS) micropatterns as a working electrode on a silk fibroin substrate. This device is ultracompliant, and can retain its integrity without loss of performance after 150 bending cycles. In addition, the whole device is shown to degrade over four weeks under enzymatic action [118]. Silk can also be used in drug delivery devices due to its biodegradability. Tao et al. [119] developed a biodegradable, remotely controllable, and implantable therapeutic device designed for infection management at a surgical site. The magnesium heater was integrated with drug-loaded silk films on a silk substrate. The drug can be released with a wireless activated heater, and the thermally-triggered drug release profiles can be controlled. It offers an expanded perspective for a restorable therapeutic medical device such as a drug delivery device.

## 5. Conclusions and Outlook

The recent development of implantable biomedical devices has been accompanied by rapid advances in both organic and inorganic functional materials. In this review, we summarized a set of three material groups, including traditional rigid materials, soft and flexible materials, and biodegradable transient materials. Silicon and silicon compounds (e.g., silicon dioxide, silicon nitride), metals, and metallic oxides have represented the first-generation materials in the development of implantable bioelectronics due to their great compatibility with well-established microfabrication processes. However, the highly stiff and rigid mechanical properties of these materials have imposed challenges in medical implantation, including damages to soft and compliant tissues and vessels. Then, soft and flexible materials were introduced, since they could improve the devices’ biocompatibility and reliability. Polymeric materials such as silicone elastomer, parylene, polyimide, and PVDF-TrFE have been widely used as substrates, while offering conformal encapsulation of electronic sensing elements in medical devices. Inorganic Si materials with open-mesh and pre-strained mechanical designs have also been attractive in flexible hybrid electronics, due to their superior electrical properties, along with their engineered stretchability. Most recently, a new category of biodegradable materials has developed to fabricate transient bioelectronics that work as temporary electrodes or supporting encapsulants. The main advantage of these materials is their natural dissolution in biofluids, while their byproducts are safely absorbed in the body. Thus, they have been widely studied to design new platforms of medical implants.

The current challenges of the transient materials are on the control of the materials’ degradation rates and the programming of dissolution triggering. Possible solutions include the addition of external stimulation (either chemical or electrical) units to the system or the integration of the lifecycle-programmed materials in the system. In addition, the underlying physics and chemistry of dissolution behaviors still need to be investigated further so that the devices can disappear after a desired lifetime. Another area to further study is the development of biodegradable integrated circuits (ICs) and chip components for high performance electronics. Replacing the existing materials and material processing steps in the circuit and device fabrication will be very challenging and require an extensive study of mechanics, surface chemistry, physical interactions, and electronics. Collectively, a comprehensive study of engineered transient materials and the integration of high-performance ICs promises next-generation, low-profile, implantable bioelectronics.

## Figures and Tables

**Table 1:** A summary of the traditional materials with applications in implantable bioelectronics.

Materials	Properties	Device Component	Applications	References
Silicon	Compatible with microfabrication	Substrate	Intraocular pressure and cardiovascular monitoring	[10,11,12,13,14,15]
Silicon	Compatible with microfabrication	Structural diaphragm	Blood pressure and shunt pressure sensor	[16,17]
Silicon oxide	High-quality factor	Structural diaphragm and substrate	Surface acoustic wave blood pressure sensor	[18,19,20]
Silicon nitride	Thermally stable	Dielectric layer	Orthopedic sensor	[21,22,23]
Silicon nitride	Thermally stable	Insulation layer	Cerebrospinal fluid flow monitoring	[24,25,26]
Stainless steel	Compatible with stents	Substrate	Capacitive pressure sensor	[27,28]

**Table 2:** Summary of organic materials with applications in biomedical devices. PDMS: polydimethylsiloxane; PVDF: polyvinylidene fluoride; LCP: liquid crystal polymer.

Materials	Properties	Device Component	Applications	References
PDMS	Low modulus, high dielectric strength, low chemical reactivity	Microfluidic channel	Pressure monitoring	[43,44]
		Dielectric layer	Pressure and oxygen sensor in blood	[45,46]
		Substrate layer	Physiological recording	[47,48,49]
Medical grade silicone	High tear strength and elasticity, transparency	Encapsulation layer	Soft contact lens sensor, intracranial and blood pressure monitoring	[50,51,52,53,54]
Parylene C	Chemical and biological inert, low water permeability and absorption	Structural diaphragm	Intraocular pressure monitoring	[55,56,57,58]
		Substrate layer	Neural electrode probe, hydrocephalus shunt occlusion detection	[59,60,61,62,63,64]
Polyimide	High heat resistance	Substrate layer	Intraocular and cardiovascular pressure monitoring	[15,65,66,67,68,69]
		Structural diaphragm	Intraocular pressure monitoring	[70,71]
PVDF	Piezoelectricity	Structural diaphragm	Intracranial and endovascular pressure monitoring	[72,73,74,75]
LCP	Low dielectric constant and low moisture absorption rate	Substrate	Intraocular pressure monitoring	[76,77,78,79]
		Encapsulation	Active intraocular pressure monitoring	[80]

**Table 3:** Summary of biodegradable materials.

Materials	Degradation Rate	Device Component	Applications	References
Magnesium	High reactivity	Electrode	Thermal therapy	[110]
Zinc and Iron	0.46 mg/(cm^2^·h)	Conductor	RF pressure sensor	[111]
Polylactic-co-Glycolic Acid (PLGA)	Several weeks	Substrate	Brain monitoring, wound healing, pressure monitoring	[113,114,115]
poly(glycerol-sebacate) (PGS)	A few months	Substrate	Cardiovascular monitoring	[116]
Silk	Several weeks	Substrate	Neural recording, drug delivery device	[86,117,118,119]
